# Impact of COVID-19 Vaccination on Patients with Non-Small Cell Lung Cancer Receiving First-Line Immune Checkpoint Inhibitor Therapy: Real-World Evidence from Romania

**DOI:** 10.3390/medicina62081588

**Published:** 2026-08-18

**Authors:** Valeriu Gheorghiță, Horia Teodor Cotan, Adriana Pistol, Cristina Maria Orlov-Slavu, Elena Tianu, Alexandra Teodora Lazar, Miruna Stanciu, Indira Radoi, Laura Mitroi, Cornelia Nițipir

**Affiliations:** 1 Faculty of Medicine, Carol Davila University of Medicine and Pharmacy, 8 Sanitary Heroes Boulevard, 050474 Bucharest, Romania; 2 “Prof. Dr. Agrippa Ionescu” Emergency Clinical Hospital, 077015 Bucharest, Romania

**Keywords:** non-small cell lung cancer, NSCLC, immune checkpoint inhibitors, immunotherapy, COVID-19 vaccination, mRNA vaccines, progression-free survival, overall survival, immune-related adverse events, real-world evidence

## Abstract

*Background*: The interaction between COVID-19 vaccination and immune checkpoint inhibitor (ICI) therapy in advanced non-small cell lung cancer (NSCLC) remains incompletely characterized. *Methods*: Retrospective cohort study of 139 patients with stage IV NSCLC treated with first-line ICI-based therapy, stratified by vaccination status and total doses received (0–1 vs. ≥2). Progression-free (PFS) and overall survival (OS) were analyzed by Kaplan–Meier and Cox regression; logistic regression explored factors associated with treatment-related toxicity. *Results*: Vaccinated patients had longer median PFS (13.0 vs. 11.0 months) and OS (29.0 vs. 24.0 months; both *p* < 0.001). In multivariable models these associations were attenuated and of borderline significance (OS HR = 0.83, 95% CI 0.69–0.99; PFS HR = 0.79, 95% CI 0.62–0.99). Outcomes were also more favorable with ≥2 doses (median PFS 14.0 vs. 12.0 months, *p* = 0.001; median OS 30.0 vs. 24.0 months, *p* < 0.001), and tumor mutational status was the strongest independent predictor of survival. In an exploratory model, ≥2 doses were associated with higher odds of any-grade toxicity (OR = 2.47, *p* = 0.037); events were predominantly low grade, with no Grade 4 or 5 toxicity. *Conclusions*: COVID-19 vaccination was associated with improved PFS and OS in stage IV NSCLC receiving first-line ICI therapy, with a dose-related pattern. The association was attenuated after adjustment for tumor biology and is hypothesis-generating. These findings support the safety and potential clinical relevance of vaccination in this population and underline the need for structured monitoring during immunotherapy. Prospective validation is required.

## 1. Introduction

The Coronavirus Disease 2019 (COVID-19) pandemic has profoundly disrupted healthcare systems worldwide and posed a particular threat to patients with cancer [1]. Individuals with non-small cell lung cancer (NSCLC) are especially vulnerable, owing to tumor-associated immune dysregulation, smoking-related comorbidities, and the immunomodulatory effects of systemic therapies [2,3]. Immune checkpoint inhibitors (ICIs) targeting programmed cell death protein-1 (PD-1), programmed death-ligand 1 (PD-L1), and cytotoxic T-lymphocyte-associated antigen 4 (CTLA-4) have transformed the management of NSCLC by restoring antitumor immune responses [4,5,6,7], yet only a fraction of patients derive sustained benefit [8], underscoring the role of intrinsic tumor alterations, endogenous antigen signaling, and the tumor microenvironment in shaping response [9].

COVID-19 vaccines, particularly mRNA-based platforms, have substantially reduced COVID-19-related morbidity and mortality [10,11]. In patients receiving ICIs, vaccination appears to be generally safe and does not substantially increase the incidence of immune-related adverse events (irAEs) [12]. ICIs nevertheless enhance T-cell activity and may improve antiviral immunity while simultaneously disrupting immune homeostasis, producing irAEs that can overlap clinically with COVID-19 manifestations such as pneumonitis and systemic inflammation—a duality that has raised questions about both the safety and the efficacy of immunotherapy during the pandemic.

Effective immunotherapy depends on coordinated activation of innate immune pathways, particularly type I interferon (IFN-I) signaling, without which adaptive responses are insufficient and ICI efficacy is reduced [13,14,15]. SARS-CoV-2 mRNA vaccines exert immunomodulatory effects beyond antiviral protection: mRNA–lipid nanoparticle (LNP) platforms induce a transient IFN-I–driven innate response that activates dendritic cells and Ly6C^+^ monocytes, enhances antigen presentation in an IFNAR1-dependent manner, and enables priming of CD8^+^ T cells. Tumor-reactive T cells consequently expand and infiltrate the tumor microenvironment, converting immunologically “cold” tumors into inflamed phenotypes [16]. In parallel, tumor cells upregulate PD-L1 as an adaptive resistance mechanism that can be overcome by checkpoint blockade, yielding synergistic antitumor activity [17].

Clinical data support these mechanistic observations. In patients with advanced NSCLC receiving ICIs, administration of COVID-19 mRNA vaccines within a defined window of immunotherapy initiation has been associated with significantly improved overall survival, even after adjustment for multiple confounders [18].

Vaccine platforms differ in their capacity to activate innate immunity. mRNA vaccines (Comirnaty^®^, Spikevax^®^) induce robust IFN-I responses through cytosolic and endosomal RNA sensing pathways including RIG-I, MDA5, and TLR7/8, producing a transient antiviral-like state. Adenoviral vector vaccines (Vaxzevria^®^, Janssen) stimulate innate immunity primarily via DNA sensing and vector-induced inflammation, generally eliciting a qualitatively different and less intense IFN-I response [19,20].

Romania provides a distinctive setting in which to examine this question. Despite a rapid rollout beginning in December 2020, the national vaccination campaign lost momentum from mid-2021 onward amid vaccine hesitancy, misinformation, and low institutional trust. Primary-course coverage plateaued at approximately 42% of the total population, compared with an EU/EEA average of approximately 73%, and first-booster uptake remained below 25% [21]. The implications of this unusual coverage pattern for the interpretation of outcome comparisons by vaccination status are considered in the Discussion.

We restricted this analysis to stage IV disease for two reasons. First, ICI therapy is standard first line in metastatic NSCLC without targetable driver alterations and is given until progression or unacceptable toxicity, so progression-free and overall survival can be interpreted as direct measures of treatment effect, without the confounding introduced by concurrent surgery or radiotherapy in earlier stages. Second, patients with metastatic malignancy were a priority group for vaccination in Romania and were vaccinated at rates exceeding the general population, yielding a cohort in which both vaccinated and unvaccinated patients were adequately represented.

Several questions remain unresolved, including the optimal timing of vaccination relative to ICI therapy, the durability of vaccine-induced immune responses, and whether vaccination may exacerbate irAEs in specific subgroups. This study therefore evaluates the association between COVID-19 vaccination and clinical outcomes in patients with stage IV NSCLC receiving first-line ICI therapy, with respect to overall survival, progression-free survival, and treatment-related toxicity.

## 2. Materials and Methods

This retrospective cohort study aimed to evaluate the impact of COVID-19 vaccination on progression-free survival (PFS) and overall survival (OS) in patients with stage IV non–small cell lung cancer (NSCLC) receiving first-line immune checkpoint inhibitor (ICI) therapy, either as monotherapy or in combination with platinum-based chemotherapy (ICI + CHT).

This study included consecutive patients with de novo stage IV NSCLC treated at the Department of Oncology of a tertiary care hospital in Bucharest, Romania, between 1 January 2022, and 1 January 2026. Treatment selection (ICI alone or ICI + CHT) was based on clinical and pathological characteristics. Patients were enrolled between January 2022 and January 2026, and the database was locked on 1 June 2026. All patients therefore had a minimum potential follow-up of five months from initiation of first-line therapy, and censoring was administrative, determined by the fixed cutoff date rather than by patient or disease characteristics.

Eligible patients were treatment-naïve, had histologically confirmed NSCLC, and had available data on COVID-19 vaccination status (including vaccine type, number of doses, and administration dates), treatment regimen, disease progression, and survival outcomes.

Exclusion criteria included a history of another active malignancy prior to or during ICI therapy, missing or unverifiable COVID-19 vaccination records, and the presence of targetable oncogenic mutations. Both mRNA and viral vector COVID-19 vaccines were considered.

Patients were categorized according to the total number of COVID-19 vaccine doses received before or during first-line therapy. The primary exposure comparison was prespecified as a binary grouping: 0–1 dose versus ≥2 doses. The timing of vaccination relative to treatment was recorded separately and analyzed as a distinct variable (within 100 days vs. more than 100 days from the date of first-line treatment initiation). Because Romania’s national vaccination campaign preceded the accrual period of this study, most patients had completed vaccination before starting first-line therapy, so that exposure status was predominantly defined at baseline.

Baseline demographic and clinical characteristics were extracted from electronic medical records, including age at diagnosis, sex, histological subtype, presence of non-targetable mutations, presence of baseline brain metastases, and tumor burden, defined as the number of organs affected by metastatic disease. Additional variables included programmed death-ligand 1 (PD-L1) expression, assessed as tumor proportion score (TPS), the occurrence of immune-related adverse events (irAEs), and comorbidities. Comorbidities were categorized into major clinical groups, including cardiovascular, pulmonary, metabolic, and renal conditions. Cardiovascular comorbidities comprised coronary artery disease, heart failure, arrhythmias, and hypertension. Pulmonary comorbidities included chronic respiratory disorders such as chronic obstructive pulmonary disease, asthma, and interstitial lung disease. Metabolic conditions encompassed diabetes mellitus, obesity, and dyslipidemia, while renal comorbidities referred primarily to chronic kidney disease or impaired renal function. Patients without any documented associated conditions were classified as having no comorbidities.

Adverse events were graded according to the National Cancer Institute Common Terminology Criteria for Adverse Events (CTCAE), version 5.0.

Histopathological diagnosis was established on tissue obtained through standard diagnostic procedures at our institution. Of the 139 patients, 94 (67.6%) were diagnosed by bronchoscopic sampling (endobronchial or transbronchial biopsy, including EBUS-TBNA), 26 (18.7%) by CT-guided transthoracic core-needle biopsy, 10 (7.2%) by biopsy of a metastatic site (pleural, lymph node, hepatic, or bone lesions), and 9 (6.5%) by surgical procedures (mediastinoscopy, video-assisted thoracoscopic surgery, or pleural biopsy). All specimens were processed and evaluated in the pathology department of the treating institution. Immunohistochemical assessment of PD-L1 expression was performed on formalin-fixed, paraffin-embedded tissue using the PD-L1 IHC 22C3 pharmDx assay (Agilent Technologies, Inc., Santa Clara, CA, USA; manufactured by Dako Denmark A/S, Glostrup, Denmark) on the Dako Autostainer Link 48 platform (Agilent Technologies, Inc., Santa Clara, CA, USA) according to the manufacturer’s instructions, and reported as the tumor proportion score (TPS). Response assessment was performed according to institutional standard of care. Baseline imaging comprised contrast-enhanced computed tomography (CT) of the thorax, abdomen and pelvis, with brain imaging by CT or magnetic resonance imaging (MRI) performed at baseline in all patients. Restaging CT was performed every 12 weeks from treatment initiation, with additional imaging obtained at any time in the presence of clinical suspicion of progression. Treatment response and disease progression were assessed by the treating oncologist in conjunction with the institutional radiology department, using Response Evaluation Criteria in Solid Tumours (RECIST) version 1.1. Imaging was evaluated as part of routine clinical practice; no independent central radiological review was performed, and iRECIST criteria were not applied.

Progression-free survival (PFS) was defined as the time from initiation of first-line treatment to radiologically confirmed disease progression or death from any cause, whichever occurred first. Overall survival (OS) was defined as the time from initiation of first-line treatment to death from any cause.

All statistical analyses were performed using IBM SPSS Statistics software version 26. Descriptive statistics were used to summarize baseline demographic and clinical characteristics. Continuous variables were reported as mean ± standard deviation or median with interquartile range (IQR), as appropriate, while categorical variables were expressed as frequencies and percentages.

Comparisons between groups (0–1 dose vs. ≥2 doses) were performed using the chi-square test or Fisher’s exact test for categorical variables and the Mann–Whitney U test for continuous variables. Linear trends across ordered groups were assessed using the linear-by-linear association test.

Survival outcomes were analyzed using the Kaplan–Meier method, and differences between groups were evaluated using the log-rank (Mantel–Cox) test. Progression-free survival (PFS) was defined as the time from treatment initiation to disease progression or death, and overall survival (OS) as the time from treatment initiation to death from any cause. Median survival times with 95% confidence intervals (CI) were reported.

To identify independent predictors of survival, multivariable Cox proportional hazards regression models were constructed, including clinically relevant covariates such as age, sex, treatment type, PD-L1 expression, metastatic burden, brain metastases, vaccination status, number of vaccine doses, and mutational status. Results were expressed as hazard ratios (HRs) with 95% confidence intervals.

To explore factors associated with treatment-related toxicity, a multivariable logistic regression model was fitted with the occurrence of any-grade treatment-related adverse event as the dependent variable. Toxicity was modelled as a binary ever/never outcome and was not standardized to person-time of treatment exposure. Results were reported as odds ratios (ORs) with 95% confidence intervals. This analysis was exploratory and was not corrected for multiple comparisons. A two-sided *p*-value < 0.05 was considered statistically significant.

## 3. Results

A total of 139 patients were included in the analysis. The mean age was 66.9 years (range 43–88, SD ± 8.0), with a predominance of male patients (*n* = 84, 60.4%), while 55 patients (39.6%) were female. The most common histological subtype was adenocarcinoma (n = 96, 69.1%), followed by squamous cell carcinoma (*n* = 37, 26.6%) and null phenotype tumors (*n* = 6, 4.3%).

At baseline, 76 patients (54.7%) presented with more than three metastatic sites, whereas 63 patients (45.3%) had fewer than three metastatic locations. Brain metastases were identified in 23 patients (16.5%), while the majority (*n* = 116, 83.5%) had no central nervous system involvement. PD-L1 expression was <1% in 89 patients (64.0%), between 1–49% in 29 patients (20.9%), and ≥50% in 21 patients (15.1%). Molecular analysis showed no actionable or specific mutations in 93 patients (66.9%), while KRAS mutations were present in 37 patients (26.6%), STK11 mutations in 7 patients (5.0%), and KEAP1 mutations in 2 patients (1.4%).

Regarding treatment, most patients received combined chemotherapy and immunotherapy (CHT + ICI) (*n* = 116, 83.5%), while 23 patients (16.5%) were treated with immunotherapy alone (ICI).

Overall, 97 patients (69.8%) were vaccinated against COVID-19, while 42 patients (30.2%) were unvaccinated. Of the vaccinated patients, 88 (90.7%) received their first dose before initiation of first-line therapy and 9 (9.3%) after, reflecting the concentration of Romania’s national vaccination campaign in 2021 relative to the accrual period of this study. Vaccination occurred within 100 days of treatment initiation in 31 patients (32.0%) and more than 100 days from treatment initiation in 66 patients (68.0%).

With regard to vaccine platform, mRNA-based vaccines were the most frequently administered (*n* = 83, 59.7% of the whole cohort), followed by viral vector vaccines (*n* = 14, 10.1%). The number of doses varied: 48 patients (34.5%) received three doses, 32 (23.0%) two doses, 15 (10.8%) one dose, and 2 (1.4%) more than three doses.

Treatment-related toxicity was absent in the majority of patients (*n* = 100, 71.9%). Among the 39 patients who experienced at least one event, the most frequent were endocrine (*n* = 19, 13.7% of the cohort), cutaneous (*n* = 7, 5.0%), and hepatic (*n* = 5, 3.6%) toxicities. Less frequent events included renal toxicity (*n* = 3, 2.2%), myositis (*n* = 3, 2.2%), rheumatologic toxicity (*n* = 1, 0.7%), and pneumonitis (*n* = 1, 0.7%). Events were predominantly low grade: according to the CTCAE classification, most were Grade 1 (*n* = 25, 64.1% of affected patients), followed by Grade 2 (*n* = 10, 25.6%). Grade 3 toxicity was uncommon (*n* = 4, 10.3%), and no Grade 4 or 5 events were reported. No immune-mediated myocarditis, pericarditis, or vasculitis was recorded.

Regarding comorbidities, 38 patients (27.3%) had no documented associated conditions. Among those with comorbidities, cardiovascular disease was the most frequent, observed in 44 patients (31.7%), followed by pulmonary conditions in 29 patients (20.9%) and metabolic disorders in 18 patients (12.9%). Renal comorbidities were less common, being reported in 10 patients (7.2%). Overall, cardiovascular and pulmonary conditions represented the predominant comorbidity burden in this cohort. This data is shown in Table 1.

Baseline characteristics were compared between vaccinated and unvaccinated patients (Table 2). The two groups were well balanced across all clinical and molecular variables assessed. There were no significant differences in sex (male, 63.9% vs. 52.4%; *p* = 0.202), histological subtype (adenocarcinoma, 72.2% vs. 61.9%; *p* = 0.474), PD-L1 expression (*p* = 0.380), molecular alterations (*p* = 0.537), presence of brain metastases (17.5% vs. 14.3%; *p* = 0.637), or metastatic burden (>3 sites, 55.7% vs. 52.4%; *p* = 0.721). Treatment allocation was virtually identical between groups, with 83.5% of vaccinated and 83.3% of unvaccinated patients receiving chemo-immunotherapy (*p* = 0.980). Any-grade treatment-related toxicity, an on-treatment outcome reported here for completeness, was recorded more frequently among vaccinated patients (34.0% vs. 14.3%, *p* = 0.017).

Patients were then stratified into 0–1 doses (n = 57) and ≥2 doses (n = 82). Most patients in both groups experienced no treatment-related adverse events (47/57 and 53/82, respectively). The distribution of toxicity across individual organ categories did not differ significantly between dose groups (χ^2^ = 8.15, *p* = 0.320), although any-grade toxicity was numerically more frequent in the ≥2-dose group (35.4% vs. 17.5%) and a significant linear trend across ordered dose categories was observed (*p* = 0.031). Patients receiving ≥2 doses were older (median 69.5 years, IQR 62–75, vs. 66.0 years, IQR 59–69.5; *p* = 0.062), with a significant linear trend toward increasing age across dose categories (*p* = 0.010).

Histology, PD-L1 expression, brain metastases, and mutational status were similarly distributed between groups. Adenocarcinoma (62/82 vs. 34/57) and KRAS mutations (25/82 vs. 12/57) were more frequent in the ≥2-dose group, whereas STK11 mutations were more common in the 0–1 dose group (5/57 vs. 2/82); none of these differences reached statistical significance (all *p* > 0.05). These data are shown in Table 3.

**Figure 1:**
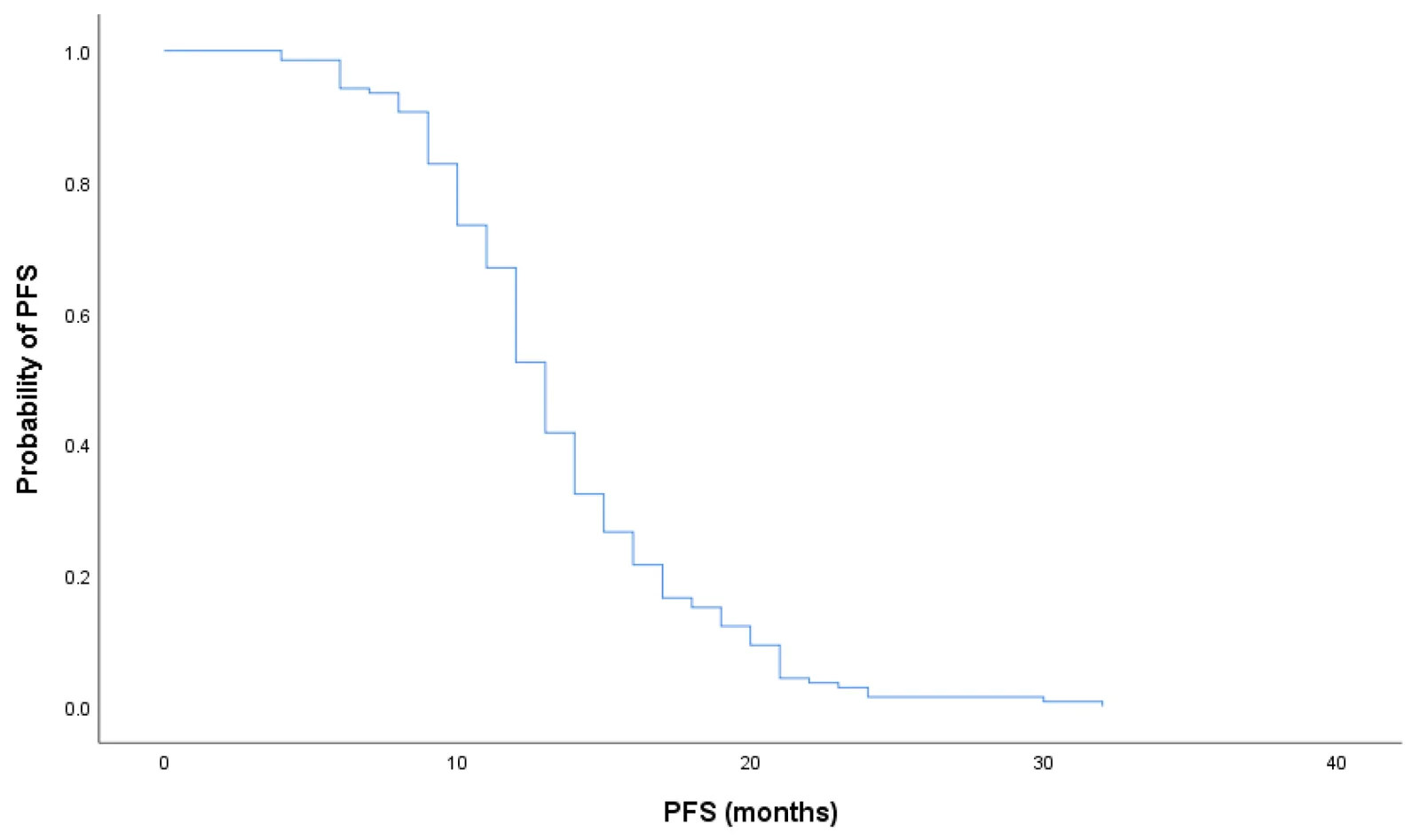
Kaplan–Meier curve showing progression-free survival (PFS) in the study cohort.

The median follow-up, estimated using the reverse Kaplan–Meier method, was 27.0 months. The median progression-free survival (PFS) for the overall cohort was 13.0 months (95% CI, 12.3–13.7) (Figure 1).

**Figure 2:**
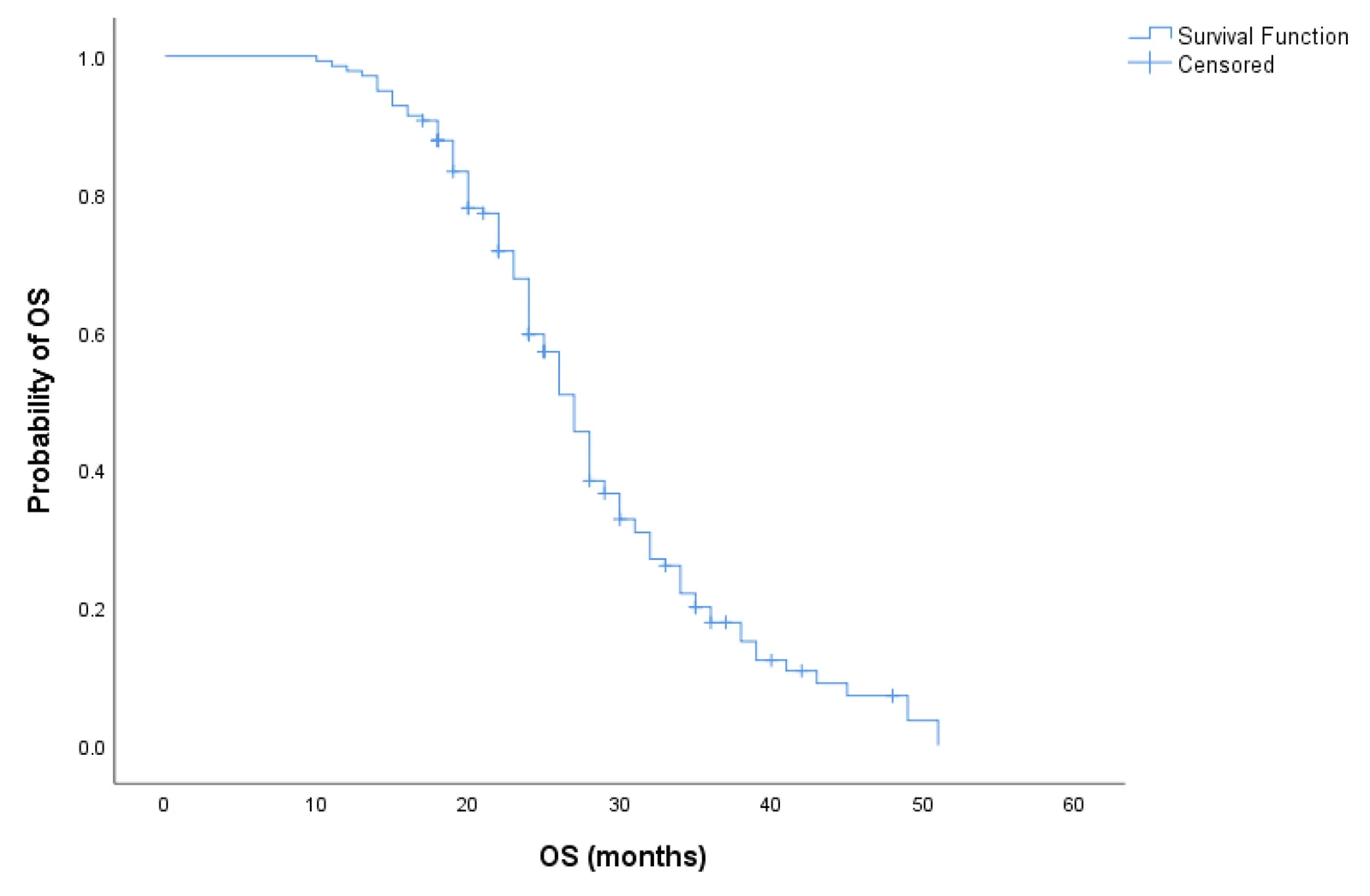
Kaplan–Meier curve showing overall survival (OS) in the study cohort.

The median overall survival (OS) was 27.0 months (95% CI, 25.6–28.4), with a mean OS of 28.3 months (95% CI, 26.5–30.0) (Figure 2).

**Figure 3:**
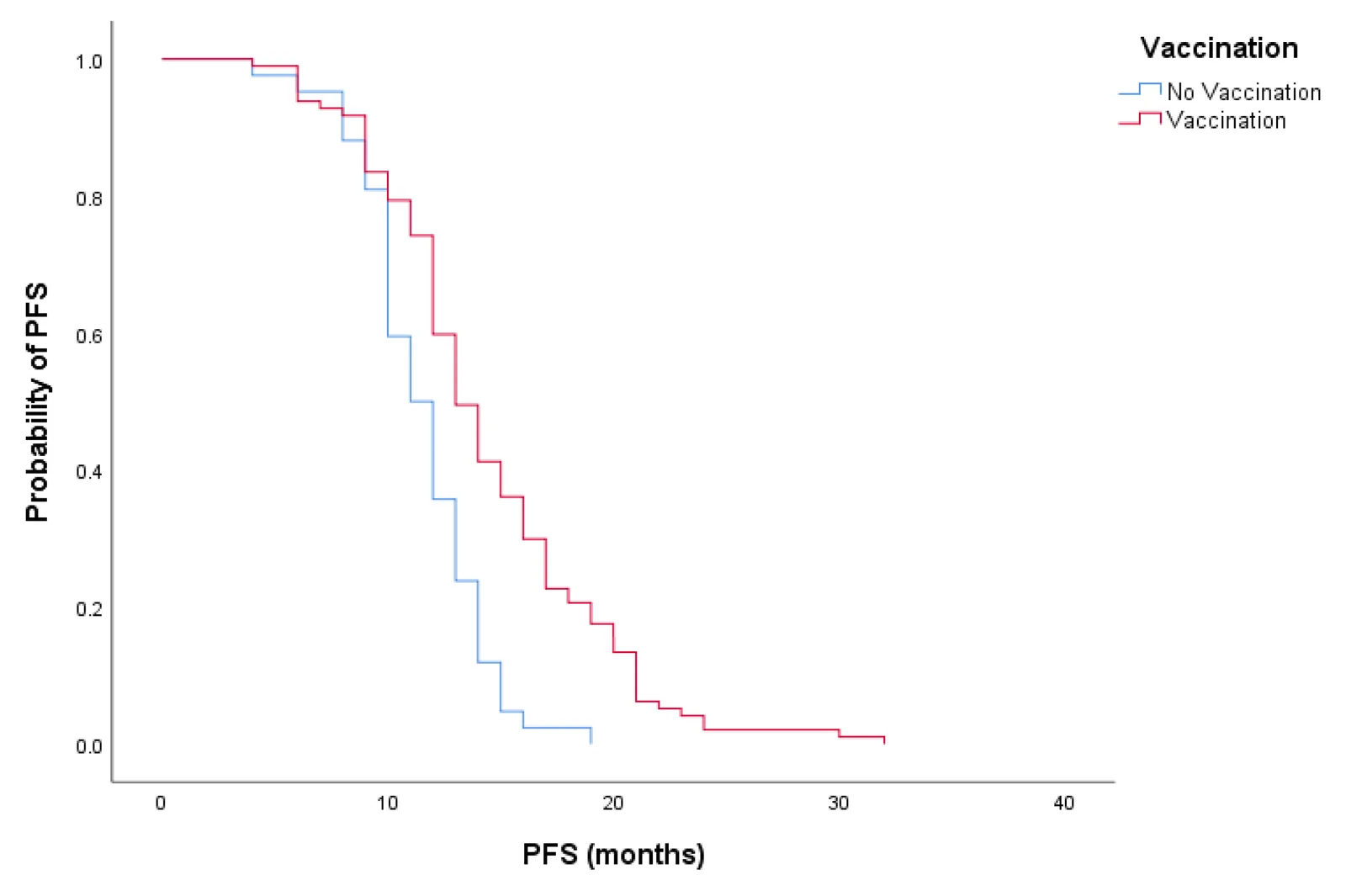
Kaplan–Meier estimates of progression-free survival (PFS) according to vaccination status.

Vaccinated patients had longer progression-free survival than unvaccinated patients. The median PFS was 13.0 months (95% CI, 11.9–14.1) in vaccinated patients versus 11.0 months (95% CI, 9.7–12.3) in unvaccinated patients (Figure 3). Survival distributions differed significantly between groups (log-rank χ^2^ = 18.06, *p* < 0.001).

**Figure 4:**
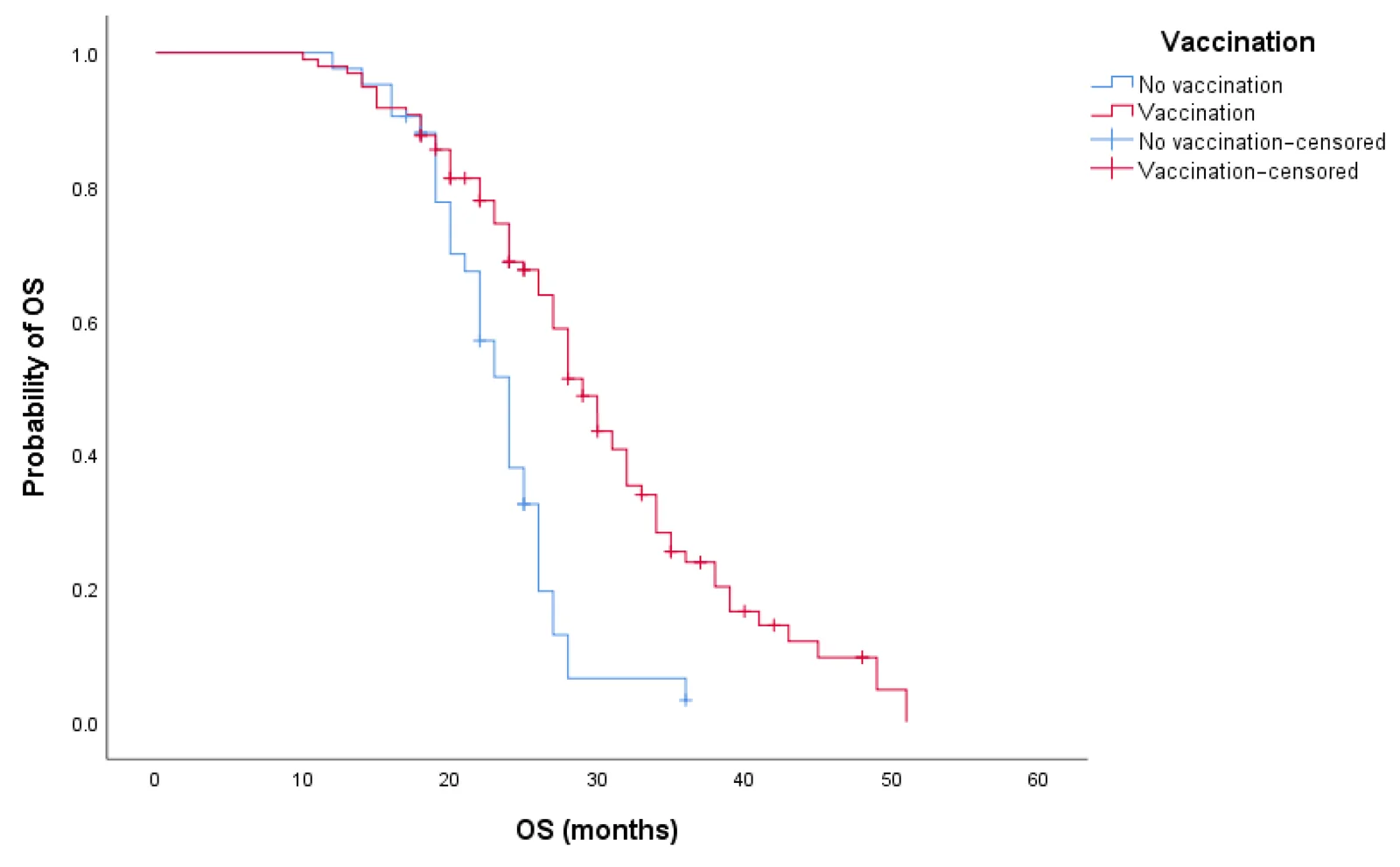
Kaplan–Meier estimates of overall survival (OS) according to vaccination status.

Vaccinated patients also had longer overall survival than unvaccinated patients. The median OS was 29.0 months (95% CI, 26.9–31.1) in vaccinated patients versus 24.0 months (95% CI, 22.4–25.6) in unvaccinated patients (Figure 4). Survival distributions differed significantly between groups (log-rank χ^2^ = 19.63, *p* < 0.001).

**Figure 5:**
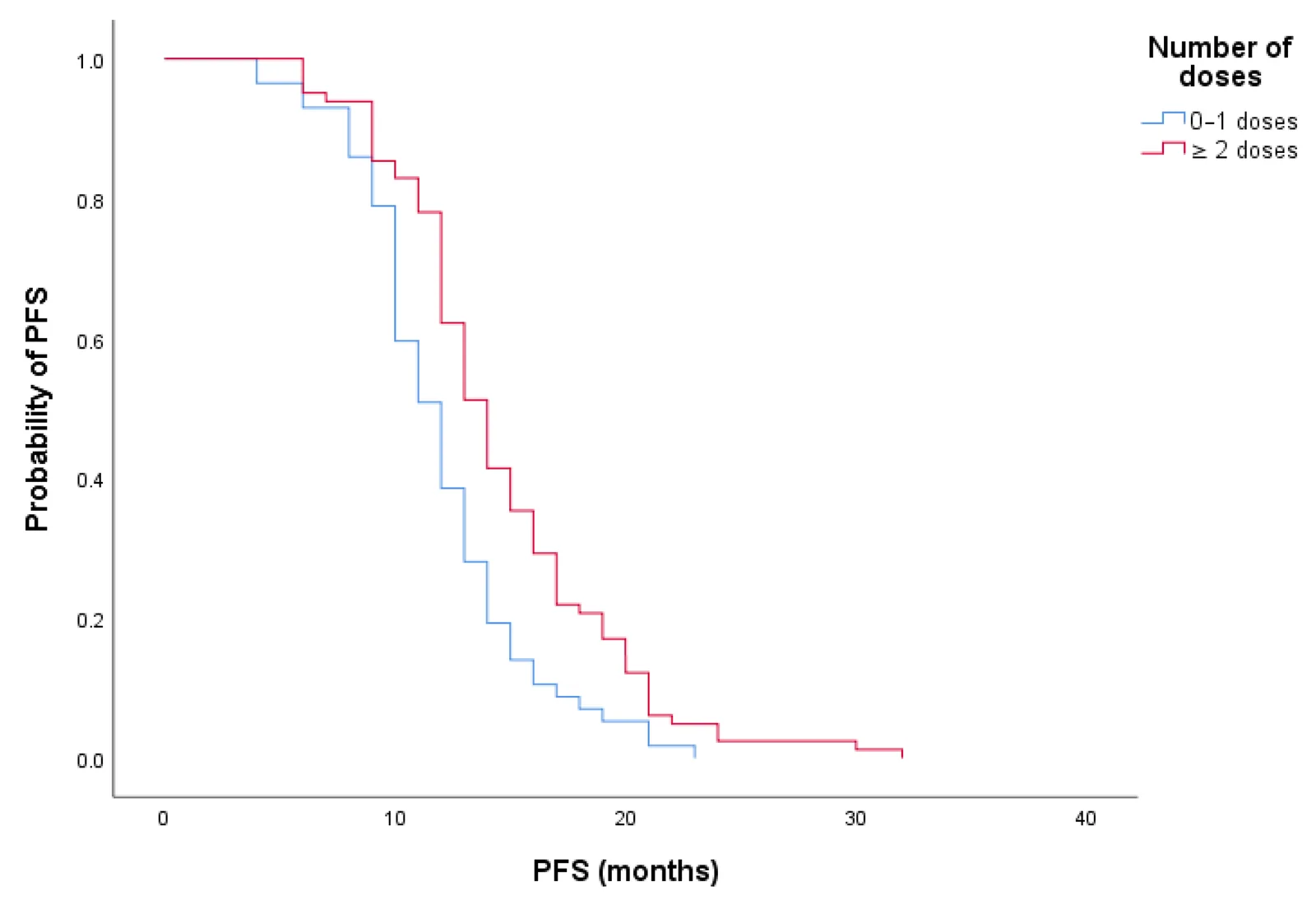
Kaplan–Meier estimates of progression-free survival (PFS) according to number of vaccine doses.

We next evaluated the association between the number of vaccine doses and survival. Patients who had received two or more doses had longer progression-free survival than those who had received 0–1 dose. The median PFS was 14.0 months (95% CI, 13.0–15.0) in the ≥2-dose group versus 12.0 months (95% CI, 10.8–13.2) in the 0–1 dose group (Figure 5). Survival distributions differed significantly between groups (log-rank χ^2^ = 11.30, *p* = 0.001).

**Figure 6:**
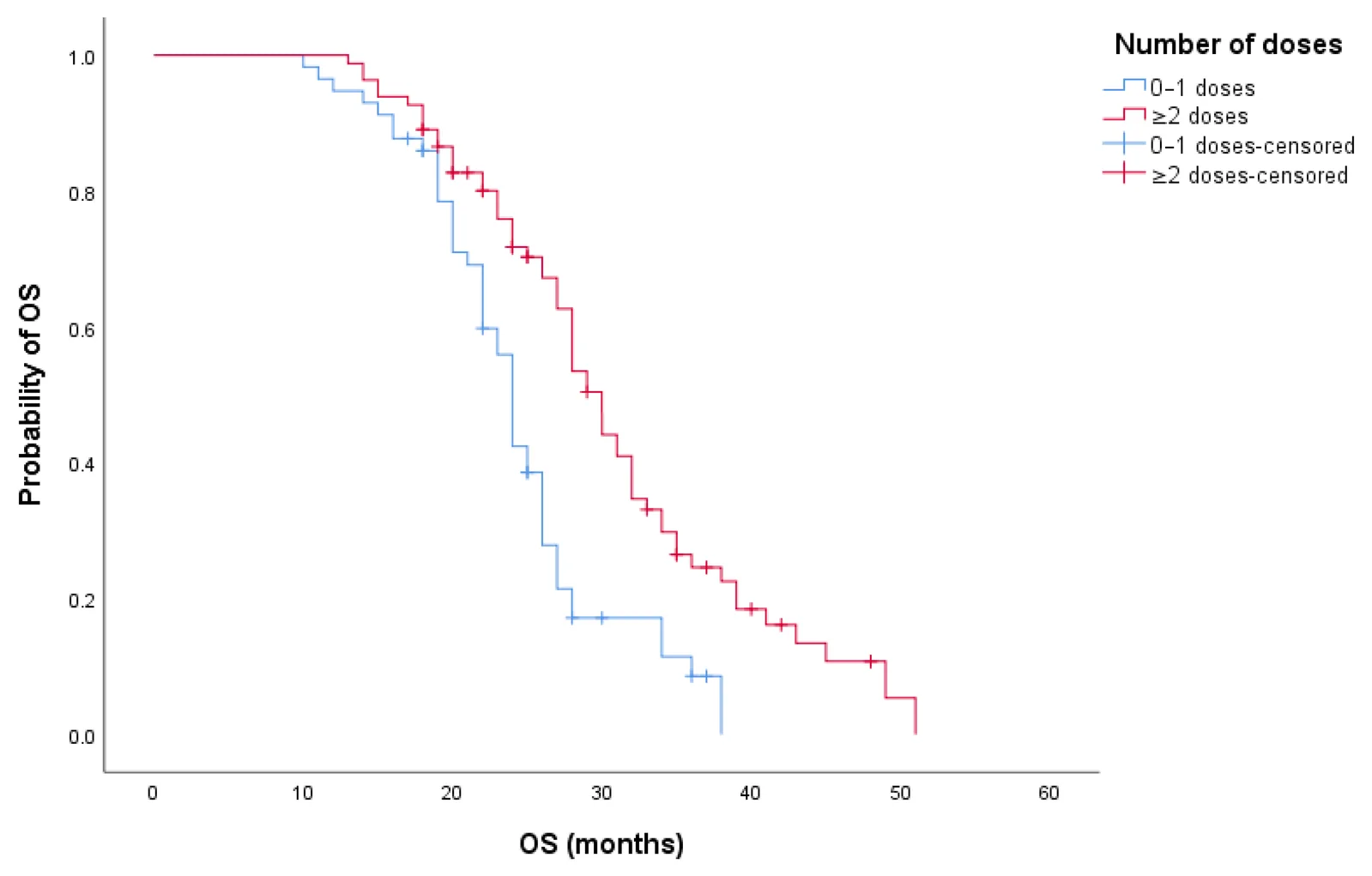
Kaplan–Meier estimates of overall survival (OS) according to number of vaccine doses.

We further evaluated the association between the number of vaccine doses and overall survival. Patients who had received two or more doses had longer overall survival than those who had received 0–1 dose. The median OS was 30.0 months (95% CI, 28.1–31.9) in the ≥2-dose group versus 24.0 months (95% CI, 23.0–25.0) in the 0–1 dose group (Figure 6). Survival distributions differed significantly between groups (log-rank χ^2^ = 15.08, *p* < 0.001).

In a multivariable Cox regression analysis, mutational status was independently associated with worse outcomes for both overall survival (OS) (HR = 2.06, 95% CI: 1.75–2.42, *p* < 0.001) and progression-free survival (PFS) (HR = 1.57, 95% CI: 1.28–1.92, *p* < 0.001).

Vaccination was associated with a reduction in the risk of death (HR = 0.83, 95% CI 0.69–0.99; *p* = 0.040) and with a comparable effect on PFS (HR = 0.79, 95% CI 0.62–0.99; *p* = 0.045). A dose-related pattern was observed: patients who had received ≥2 doses had improved OS (HR = 0.71, 95% CI 0.49–0.95; *p* = 0.040), whereas the corresponding estimate for PFS did not reach statistical significance (HR = 0.91, 95% CI 0.61–1.10; *p* = 0.060).

A higher metastatic burden was associated with worse OS (HR = 1.31, 95% CI 1.15–1.64; *p* = 0.035), whereas male sex showed a borderline association (HR = 1.13, 95% CI 0.99–1.28; *p* = 0.055) and the presence of brain metastases was not independently associated with survival (HR = 1.51, 95% CI 0.81–2.82; *p* = 0.190). The timing of vaccination relative to treatment initiation was not associated with survival outcomes: patients vaccinated within 100 days of treatment initiation had similar overall survival (HR = 1.23, 95% CI 0.56–1.74; *p* = 0.233) and progression-free survival (HR = 1.05, 95% CI 0.43–1.64; *p* = 0.345) compared with those vaccinated more than 100 days from treatment initiation.

No significant associations were observed for vaccine platform, treatment modality, PD-L1 expression, or the occurrence of treatment-related toxicity, which was not associated with either OS (HR = 1.34, 95% CI 0.84–2.14; *p* = 0.217) or PFS (HR = 1.42, 95% CI 0.85–1.76; *p* = 0.197). These data are shown in Table 4.

An exploratory multivariable logistic regression model was fitted to identify factors associated with the occurrence of any-grade treatment-related toxicity. After adjustment for age, treatment regimen, PD-L1 expression, and metastatic burden, receipt of ≥2 vaccine doses was associated with higher odds of recorded toxicity compared with 0–1 dose (OR = 2.47, 95% CI 1.06–5.76; *p* = 0.037). The treatment regimen carried the largest point estimate in the model without reaching significance (CHT + ICI vs. ICI alone, OR = 4.94, 95% CI 0.97–25.24; *p* = 0.055), and no association was observed for age, PD-L1 expression, or metastatic burden. These data are shown in Table 5.

## 4. Discussion

In this retrospective cohort study, we examined the association between COVID-19 vaccination and clinical outcomes in patients with stage IV NSCLC receiving first-line immune checkpoint inhibitor (ICI) therapy. Vaccinated patients had longer progression-free survival (PFS) and overall survival (OS) in unadjusted analyses, with a dose-related pattern favoring patients who had received two or more doses; these differences were attenuated after adjustment for tumor biology and treatment-related variables. Any-grade treatment-related toxicity was recorded more frequently among more extensively vaccinated patients, but this difference was confined to low-grade events, was not accompanied by any difference in the pattern or severity of toxicity, and, as discussed below, is open to several non-causal explanations.

These findings are consistent with emerging evidence suggesting that COVID-19 vaccination may potentiate antitumor immunity in patients treated with ICIs. A recent meta-analysis including 4929 patients treated with ICIs demonstrated that COVID-19 vaccination was associated with significantly improved PFS (pooled HR = 0.66) and OS (pooled HR = 0.51) compared with unvaccinated patients [18]. Similarly, our cohort demonstrated significantly prolonged survival among vaccinated patients, supporting the hypothesis that vaccine-induced immune activation may enhance responsiveness to immune checkpoint blockade. Notably, no deaths in our cohort were attributable to COVID-19 infection. The survival difference observed between vaccinated and unvaccinated patients therefore cannot be explained by the vaccine’s direct protective effect against fatal infection, which represents the most parsimonious alternative explanation for associations of this kind reported elsewhere.

**Figure 7:**
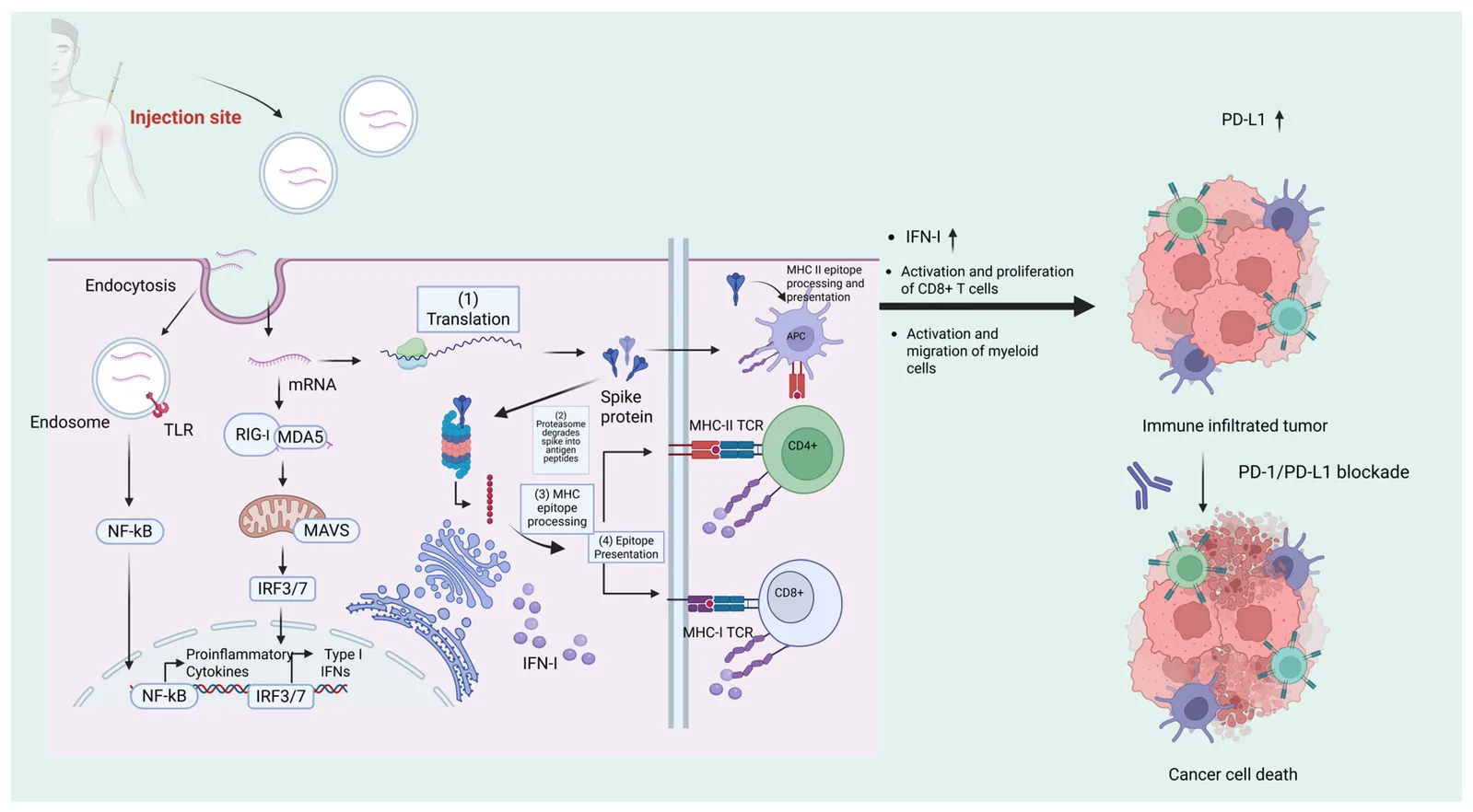
Mechanistic overview of mRNA vaccine–induced immune activation and interaction with PD-1/PD-L1 blockade.

Our findings are consistent with translational and clinical data reported by Grippin et al. [16], demonstrating that SARS-CoV-2 mRNA vaccines may enhance sensitivity to PD-1/PD-L1 blockade through type I interferon–mediated immune activation. In that study, vaccination administered within 100 days of ICI initiation was associated with improved survival outcomes in patients with advanced NSCLC. Mechanistically, mRNA–lipid nanoparticle platforms promote dendritic cell activation, enhance CD8^+^ T-cell priming, increase tumor immune infiltration, and upregulate PD-L1 expression within the tumor microenvironment (Figure 7).

Legend: mRNA vaccines are taken up by antigen-presenting cells, leading to spike protein expression, antigen presentation, and activation of innate immune pathways, including type I interferon signaling. This promotes T-cell activation and immune infiltration of the tumor microenvironment, with increased PD-L1 expression. In combination with PD-1/PD-L1 blockade, this enhances antitumor immunity and tumor cell killing.

More broadly, mRNA vaccines may transiently modulate the tumor–immune interface, promoting a shift toward a more inflamed and immunologically active phenotype, thereby potentially enhancing responsiveness to immune checkpoint blockade [22].

Our findings, and in particular the more favorable outcomes observed in patients who had received multiple vaccine doses, are biologically consistent with these proposed mechanisms. Patients who had received two or more doses had longer PFS and OS than those who had received 0–1 dose. In multivariable Cox regression this dose-related association was attenuated, remaining nominally significant for OS (HR = 0.71; *p* = 0.040) but not for PFS (HR = 0.91; *p* = 0.060), although the direction of effect was consistent across analyses. This attenuation may reflect limited statistical power arising from the relatively small sample size and the inclusion of correlated variables, namely vaccination status and dose number, within the same model. The persistence of this pattern is nevertheless compatible with accumulating evidence that repeated mRNA vaccination enhances both innate and adaptive immune responses, including sustained type I interferon signaling, dendritic cell activation, and T-cell priming [16,19,23]. Translational and clinical studies have shown that SARS-CoV-2 mRNA vaccines can sensitize tumors to PD-1/PD-L1 blockade through immune activation pathways [16], and recent work suggests that these vaccines may transiently reshape the tumor–immune interface, converting immunologically cold tumors into more inflamed and responsive phenotypes [22]. Together, these observations support the biological plausibility of a cumulative immunostimulatory effect of repeated vaccine exposure, which our data are consistent with but cannot establish.

Receipt of two or more vaccine doses was also associated with higher odds of any-grade treatment-related toxicity in the exploratory logistic model (OR = 2.47, 95% CI 1.06–5.76). This finding requires careful interpretation, and several considerations argue against reading it as evidence that COVID-19 vaccination causes immune-related toxicity during checkpoint blockade.

First, the estimate is statistically fragile. The lower bound of the confidence interval lies immediately above unity, the model was fitted to only 39 events across five covariates below the conventional threshold of ten events per variable required for stable logistic regression estimates and no correction for multiple comparisons was applied. Consistent with this fragility, the distribution of toxicity across individual organ categories did not differ significantly between dose groups (*p* = 0.320), and the occurrence of toxicity was not associated with either OS or PFS in the Cox models (Table 4).

Second, and most importantly, toxicity was analyzed as a binary ever/never outcome, without accounting for differential time at risk. Patients who had received two or more doses remained progression-free for a median of 14.0 months, compared with 12.0 months in the 0–1 dose group, and therefore accumulated a longer period of active immunotherapy during which an adverse event could occur and be documented. Because the cumulative probability of experiencing at least one adverse event increases with the duration of treatment, any comparison of cumulative incidence that is not standardized to person-time will systematically favor the group with the shorter treatment duration. An incidence-rate analysis expressed per patient-year of ICI exposure would be required to determine whether a genuine excess risk exists, and this represents a priority for future work.

Third, ascertainment is likely to have differed between groups. Vaccinated patients had, by definition, more frequent contact with health services, and 64.1% of the events recorded in this cohort were Grade 1 precisely the category most sensitive to differences in the intensity of clinical observation and documentation.

Fourth, residual confounding cannot be excluded. Patients in the ≥2-dose group were older, with a significant trend across dose categories, and Eastern Cooperative Oncology Group performance status, smoking history, and comorbidity indices were not available for adjustment. Within the same model, the treatment regimen carried by far the largest point estimate (CHT + ICI vs. ICI alone, OR = 4.94), indicating that regimen-related toxicity is a dominant and incompletely captured determinant of the outcome.

Finally, the temporal structure of the exposure makes a direct causal contribution implausible. Of the vaccinated patients, 90.7% received their first dose before starting immunotherapy, and 68.0% were vaccinated more than 100 days from treatment initiation, whereas the innate immune activation induced by mRNA vaccines peaks within days and resolves within weeks. Immune-related adverse events, by contrast, typically emerge weeks to months after ICI initiation. A pharmacodynamic interaction between a vaccine administered long before therapy and toxicity arising during therapy therefore has no established mechanistic basis.

Taken together with the clinical profile of the events observed predominantly Grade 1–2, no Grade 4 or 5 events, no immune-mediated myocarditis, pericarditis, or vasculitis, and no toxicity-related deaths these considerations indicate that our data do not demonstrate an excess of clinically meaningful toxicity attributable to vaccination. They are consistent with the substantial body of prospective evidence, including the Vax-On-Third study, showing that COVID-19 vaccination, including booster doses, does not compromise the safety of immune checkpoint inhibitor therapy [12,24]. The association reported here should therefore be regarded as an unconfirmed signal arising from a small exploratory model, and not as a safety finding. We note in addition that the occurrence of irAEs has itself been associated with greater ICI efficacy in several settings [25,26], so that a higher rate of documented low-grade events in a group with longer treatment duration is not, in itself, an adverse prognostic observation.

Prospective studies incorporating immune monitoring and person-time–based analyses of adverse events are needed to characterize the relationship between vaccine-induced immune activation, therapeutic efficacy, and toxicity.

In multivariable analyses, mutational status emerged as the strongest independent predictor of both OS and PFS. Patients harboring KRAS, STK11, or KEAP1 alterations demonstrated significantly worse outcomes, which is consistent with existing literature showing that STK11 and KEAP1 mutations are associated with immune resistance, reduced T-cell infiltration, and inferior responses to ICIs [27,28,29]. The strong prognostic influence of tumor biology likely explains why the independent effect of vaccination was attenuated in adjusted models despite clear differences in Kaplan–Meier analyses.

Our findings are further supported by recent large-scale real-world data from a French nationwide cohort study [30] including over 30,000 patients treated with PD-(L)1 inhibitors, in which exposure to a COVID-19 mRNA vaccine within 90 days of treatment initiation was associated with a modest but statistically significant improvement in overall survival (weighted HR = 0.91, 95% CI 0.88–0.93). This benefit was consistent across tumor types, including NSCLC, supporting the generalizability of a potential interaction between vaccination and immune checkpoint blockade.

In addition to observational and translational evidence, ongoing clinical trials are actively investigating the combination of mRNA vaccines with immune checkpoint inhibitors in NSCLC. For example, the mRNA-4359 + pembrolizumab trial (NCT05533697) [31] is evaluating the safety and efficacy of a personalized mRNA vaccine in combination with PD-1 blockade in patients with advanced or metastatic NSCLC. These studies are based on the rationale that mRNA platforms can enhance tumor-specific immune priming, thereby improving the effectiveness of checkpoint inhibition. Unlike COVID-19 vaccines, which provide non-specific immune stimulation, these next-generation mRNA vaccines are designed to encode tumor-associated antigens, potentially leading to a more targeted and durable antitumor response.

That said, two of our findings deserve closer examination before firm conclusions are drawn. The first is the lack of association between vaccination timing and survival outcomes—a result that sits in apparent tension with the analysis by Grippin et al., in which vaccination within 100 days of ICI initiation was the central exposure definition [16]. Several factors may explain this discrepancy. The 100-day window has recently been questioned on methodological grounds: a target trial emulation re-analysis of the original dataset found no survival benefit after correction for immortal time bias and for differences in the definition of follow-up, raising the possibility that the timing effect in the original analysis was at least partly analytical in origin [32]. Beyond this, the biology of mRNA vaccine-induced interferon signaling, which peaks within days and resolves within weeks, makes it difficult to argue for a mechanistically meaningful window as wide as 100 days. Our dose-related findings point instead toward cumulative immune priming as the more likely operative mechanism. This interpretation is consistent with the Vax-On-Third study, a prospective Italian cohort in which booster vaccination around ICI initiation conferred no overall survival benefit but did improve outcomes specifically in patients with high PD-L1 expression receiving ICI monotherapy, suggesting that the interaction may depend more on tumor immunogenicity than on timing [24]. The mechanistic arm of the same programme, the Vax-On-Third-Profile substudy, found that booster doses were followed by meaningful increases in natural killer cell counts that independently predicted both reduced treatment failure and longer overall survival [33]—a durable cellular effect that is unlikely to depend on a narrow pre-treatment window. Taken together, these data suggest that what matters may be the cumulative depth of vaccine-induced immune engagement across multiple doses, rather than the timing of any single dose relative to ICI initiation.

The second finding that warrants comment is the absence of a survival difference between recipients of mRNA and adenoviral vector vaccines (HR = 0.89, *p* = 0.545). The mechanistic case for a vaccine–ICI interaction rests heavily on mRNA-specific type I interferon signaling [16], which might lead one to expect a platform-dependent clinical effect. In practice, however, the immunological boundary between platforms is less sharp than is often assumed: adenoviral vectors also activate IFN-I-mediated innate-to-adaptive signaling and generate durable T-cell responses [34,35]. More importantly, with only 14 patients in the vector vaccine group, this comparison was never adequately powered to detect a difference. Grippin et al. similarly observed no differential effect across manufacturers in a cohort nearly ten times the size of ours [16]. Our null result should therefore not be taken as evidence of true equivalence between platforms; it reflects the limits of what a small, imbalanced subgroup analysis can reliably show.

During the COVID-19 pandemic, the potential role and clinical impact of COVID-19 vaccination in patients with cancer became a major area of concern, particularly in Romania, where vaccine hesitancy was among the highest reported in the European Union. The relevance of the present study is further strengthened by its focus on a high-risk population of oncology patients, characterized by increased susceptibility to severe COVID-19 and by a substantial burden of comorbidities. National data from the Romanian National Institute of Public Health indicated that cancer was among the leading risk factors for COVID-19-related death, ranking fourth after cardiovascular disease, chronic kidney disease, and diabetes mellitus. In that analysis, cancer was associated with more than a twofold increase in the odds of death [OR 2.1876, 95% CI 1.5049–3.1800] compared with individuals in whom these conditions were absent [36].

Romania provides a distinctive setting in which to examine this question. Despite an initially rapid rollout beginning in December 2020, which prioritized healthcare workers and clinically vulnerable groups, the national COVID-19 vaccination campaign lost momentum from mid-2021 onward amid widespread vaccine hesitancy, misinformation, and low trust in public institutions. Cumulative primary-course coverage plateaued at approximately 42% of the total population, compared with an EU/EEA average of approximately 73%, while first-booster uptake remained below 25%, placing Romania among the lowest-coverage member states in the European Union [21]. This context is directly relevant to the interpretation of studies conducted in this population. In most healthcare systems with high uptake, unvaccinated individuals represent a self-selected minority in whom non-vaccination may correlate with poorer health-seeking behavior, reduced healthcare access, or worse baseline status—factors that confound any comparison of clinical outcomes by vaccination status. In Romania, by contrast, non-vaccination was the majority behavior and was driven predominantly by societal rather than individual health-related determinants, which may attenuate this source of confounding. At the same time, patients with cancer were designated a priority group and received targeted counselling, which likely accounts for the higher vaccination rate observed in oncology cohorts than in the general population.

Our study has several clinical implications. First, the data are consistent with the safety of COVID-19 vaccination in patients receiving ICIs and provide no evidence that vaccination compromises oncological outcomes. Second, the association between repeated vaccination and longer survival raises the hypothesis that vaccine-mediated innate immune stimulation might act as an adjunctive immunomodulatory strategy, a hypothesis that requires prospective testing. Third, toxicity in this cohort was predominantly low grade and manageable, no severe or fatal treatment-related events occurred, and the occurrence of toxicity was not associated with worse survival; these observations support current recommendations that patients with advanced NSCLC receiving immunotherapy should be offered COVID-19 vaccination in accordance with national immunization programmes.

Several limitations should be acknowledged. The retrospective design introduces the possibility of selection bias and residual confounding. Vaccinated patients may have differed from unvaccinated individuals in terms of healthcare access, performance status, or comorbidity burden. Notably, Eastern Cooperative Oncology Group performance status was not consistently documented in the source records and could not be included in our multivariable models; as performance status is among the strongest prognostic determinants in advanced NSCLC, its omission represents an important source of residual confounding. Although multivariable analyses were performed, unmeasured confounders could not be excluded. Additionally, the relatively limited cohort size may have reduced the power of adjusted analyses.

Heterogeneity in the timing of vaccination relative to treatment initiation also represents a potential confounding factor, particularly in view of prior evidence suggesting that vaccination within a narrow temporal window around ICI initiation may maximize any immunological interaction.

Our study collected no immunological or laboratory correlates—including cytokine profiles, lymphocyte subsets, interferon signatures, or natural killer cell counts—and we are therefore unable to substantiate any mechanistic interpretation of the observed associations. In particular, the association between vaccine dose number and recorded treatment-related toxicity cannot be attributed to enhanced immune activation on the basis of clinical data alone. As detailed above, it may equally reflect the longer duration of immunotherapy exposure in more extensively vaccinated patients, differential surveillance and documentation of low-grade events, or residual confounding. A time-to-event or incidence-rate analysis of toxicity standardized to person-time of treatment was not feasible with the available data and should be incorporated in future studies.

Vaccination and dose category were modelled as time-fixed exposures. For most patients (90.7% of those vaccinated), vaccination preceded treatment initiation and exposure was therefore defined at baseline, so that no immortal time was generated. In the minority of patients who received doses after starting therapy, person-time accrued before those doses was attributed to the higher-dose category, and a residual susceptibility to immortal time bias remains. A time-dependent Cox model would address this directly and represents a priority for future analyses, particularly in cohorts where vaccination occurs predominantly during rather than before immunotherapy.

An important limitation concerns unmeasured confounding. Eastern Cooperative Oncology Group performance status, smoking history, body mass index, and socioeconomic indicators were not consistently recorded in the source documentation and could not be included in our multivariable models.

Finally, response was assessed by investigators using RECIST 1.1 without independent central review, and iRECIST criteria were not applied; atypical response patterns such as pseudoprogression may therefore have been recorded as progression events. Imaging intervals reflected routine clinical practice rather than a protocol-mandated schedule, which may introduce ascertainment bias into progression-free survival estimates.

Despite these limitations, our findings add to the growing body of evidence indicating that COVID-19 vaccination can be administered safely to patients receiving immune checkpoint blockade and may be associated with immunomodulatory effects of potential clinical relevance. Prospective studies and randomized clinical trials are required to validate these observations, to define the optimal timing and number of vaccine doses, and to elucidate the underlying immunological mechanisms.

## 5. Conclusions

In this real-world cohort of patients with stage IV NSCLC receiving first-line immune checkpoint inhibitor therapy, COVID-19 vaccination was associated with longer progression-free and overall survival, with a dose-related pattern favoring patients who had received two or more doses. This association was attenuated after multivariable adjustment, in which tumor mutational status emerged as the strongest independent determinant of survival, and should therefore be regarded as hypothesis-generating rather than confirmatory.

Overall, these findings support the safety and the potential clinical relevance of COVID-19 vaccination in patients undergoing immune checkpoint blockade, a population at substantially increased risk of severe COVID-19, and underline the importance of structured clinical monitoring throughout immunotherapy. Prospective studies incorporating immunological correlates are needed to confirm these observations and to define the optimal timing and number of vaccine doses.

## Figures and Tables

**Table 1:** Baseline Demographic, Tumor, Treatment, and Vaccination Characteristics of Patients.

Variable	Category	*n* (%)
Age (years)	Mean ± SD	66.9 ± 8.0
Gender	Male	84 (60.4)
	Female	55 (39.6)
Histology	Adenocarcinoma	96 (69.1)
	Squamous	37 (26.6)
	Other	6 (4.3)
Metastatic burden	≤3 sites	63 (45.3)
	>3 sites	76 (54.7)
Brain metastases	No	116 (83.5)
	Yes	23 (16.5)
PD-L1 expression	<1%	89 (64.0)
	1–49%	29 (20.9)
	≥50%	21 (15.1)
Mutational status	None specific	93 (66.9)
	KRAS	37 (26.6)
	STK11	7 (5.0)
	KEAP1	2 (1.4)
Treatment	CHT + ICI	116 (83.5)
	ICI alone	23 (16.5)
Vaccination status	No	42 (30.2)
	Yes	97 (69.8)
Timing of vaccination relative to treatment initiation *	<100 days	31 (32.0)
	>100 days	66 (68.0)
Number of doses	none	42 (30.2)
	1 dose	15 (10.8)
	2 doses	32 (23.0)
	3 doses	48 (34.5)
	>3 doses	2 (1.4)
Toxicities	None	100 (71.9)
	Endocrinologic	19 (13.7)
	Cutaneous	7 (5.0)
	Hepatic	5 (3.6)
	Renal	3 (2.2)
	Myositis	3 (2.2)
	Rheumatologic	1 (0.7)
	Pneumonitis	1 (0.7)
CTCAE grade (evaluable patients)	Grade 1	25 (64.1)
	Grade 2	10 (25.6)
	Grade 3	4 (10.3)
Comorbidities	None	38 (27.3)
	Cardiac	44 (31.7)
	Pulmonary	29 (20.9)
	Metabolic	18 (12.9)
	Renal	10 (7.2)

Percentages for timing of vaccination are calculated among vaccinated patients (n = 97) *. Abbreviations: n, number of patients; SD, standard deviation; PD-L1, programmed death-ligand 1; KRAS, Kirsten rat sarcoma viral oncogene homolog; STK11, serine/threonine kinase 11; KEAP1, Kelch-like ECH-associated protein 1; CHT, chemotherapy; ICI, immune checkpoint inhibitor; CHT + ICI, combination of chemotherapy and immune checkpoint inhibitor; CTCAE, Common Terminology Criteria for Adverse Events; mRNA, messenger ribonucleic acid.

**Table 2:** Baseline characteristics of vaccinated and unvaccinated patients.

Characteristic	Unvaccinated (*n* = 42)	Vaccinated (*n* = 97)	*p*
Sex			0.202
Female	20 (47.6)	35 (36.1)	
Male	22 (52.4)	62 (63.9)	
Histology			0.474
Adenocarcinoma	26 (61.9)	70 (72.2)	
Squamous	14 (33.3)	23 (23.7)	
Null phenotype	2 (4.8)	4 (4.1)	
PD-L1 expression			0.380
<1%	28 (66.7)	61 (62.9)	
1–49%	6 (14.3)	23 (23.7)	
≥50%	8 (19.0)	13 (13.4)	
Molecular alterations			0.537
None specific	30 (71.4)	63 (64.9)	
KRAS	9 (21.4)	28 (28.9)	
STK11	3 (7.1)	4 (4.1)	
KEAP1	0 (0.0)	2 (2.1)	
Brain metastases			0.637
Absent	36 (85.7)	80 (82.5)	
Present	6 (14.3)	17 (17.5)	
Metastatic burden			0.721
≤3 sites	20 (47.6)	43 (44.3)	
>3 sites	22 (52.4)	54 (55.7)	
Treatment regimen			0.980
CHT + ICI	35 (83.3)	81 (83.5)	
ICI alone	7 (16.7)	16 (16.5)	
Any treatment-related toxicity (on-treatment outcome)			0.017
No	36 (85.7)	64 (66.0)	
Yes	6 (14.3)	33 (34.0)	

Values are n (%) unless otherwise stated. CHT, chemotherapy; ICI, immune checkpoint inhibitor; SD, standard deviation. *p*-values from Pearson chi-square test for categorical variables and independent-samples *t*-test for age. Any-grade treatment-related toxicity is an on-treatment outcome rather than a baseline characteristic and is shown here for completeness.

**Table 3:** Baseline Clinical and Tumor Characteristics According to Vaccination Dose Groups.

Variable	Category	0–1 Doses (n = 57)	≥2 Doses (n = 82)	*p*-Value
Age	-	66 (59–69.5)	69.5 (62–75)	0.062 *
Histology	Adenocarcinoma	34 (59.6%)	62 (75.6%)	0.132
	Squamous	20 (35.1%)	17 (20.7%)	
	Other	3 (5.3%)	3 (3.7%)	
Brain metastases	No	47 (82.5%)	69 (84.1%)	0.792
	Yes	10 (17.5%)	13 (15.9%)	
PD-L1	<1%	37 (64.9%)	52 (63.4%)	0.637
	1–49%	10 (17.5%)	19 (23.2%)	
	≥50%	10 (17.5%)	11 (13.4%)	
Toxicities	None	47 (82.5%)	53 (64.6%)	0.320
	Endocrinologic	7 (12.3%)	12 (14.6%)	
	Renal	0 (0.0%)	3 (3.7%)	
	Cutaneous	1 (1.8%)	6 (7.3%)	
	Rheumatologic	0 (0.0%)	1 (1.2%)	
	Myositis	1 (1.8%)	2 (2.4%)	
	Pneumonitis	0 (0.0%)	1 (1.2%)	
	Hepatic	1 (1.8%)	4 (4.9%)	
Mutations	None	40 (70.2%)	53 (64.6%)	0.148
	KRAS	12 (21.1%)	25 (30.5%)	
	STK11	5 (8.8%)	2 (2.4%)	
	KEAP1	0 (0.0%)	2 (2.4%)	

* Likelihood ratio *p* = 0.007; linear trend *p* = 0.010. Abbreviations: PD-L1, programmed death-ligand 1; KRAS, Kirsten rat sarcoma viral oncogene homolog; STK11, serine/threonine kinase 11; KEAP1, Kelch-like ECH-associated protein 1. Age was compared using the Mann–Whitney U test and is reported as median (interquartile range). The *p*-value for toxicity refers to the distribution across individual toxicity categories.

**Table 4:** Multivariable Cox Regression Analysis of Factors Associated with Overall Survival (OS) and Progression-Free Survival (PFS).

Test Variables	OS HR (95% CI)	*p*-Value	PFS HR (95% CI)	*p*-Value
Vaccination no (ref.)/yes	0.83 (0.69–0.99)	0.040	0.79 (0.62–0.99)	0.045
Type of Vaccine vector/mRNA (ref.)	0.89 (0.37–1.16)	0.545	0.94 (0.58–1.51)	0.798
Doses ≥ 2/0–1 (ref.)	0.71 (0.49–0.95)	0.040	0.91 (0.61–1.10)	0.060
Any toxicity (ref.)/no toxicity	1.34 (0.84–2.14)	0.217	1.42 (0.85–1.76)	0.197
Gender female (ref.)/male	1.13 (0.99–1.28)	0.055	1.68 (0.76–2.45)	0.130
Treatment ICI (ref.)/CHT + ICI	1.32 (0.84–1.59)	0.214	1.51 (0.77–1.84)	0.235
Metastatic burden ≤ 3 metastatic sites(ref.)/>3 metastatic sites	1.31 (1.15–1.64)	0.035	0.99 (0.66–1.49)	0.175
Brain metastases none (ref.)/present	1.51 (0.814–2.82)	0.190	1.28 (0.68–2.41)	0.236
Mutational status no mutations (ref.)/mutations	2.06 (1.75–2.42)	<0.001	1.57 (1.28–1.92)	<0.001
Vaccination <100 days/>100 days (ref)	1.23 (0.56–1.74)	0.233	1.05 (0.43–1.64)	0.345

Abbreviations: HR, hazard ratio; CI, confidence interval; OS, overall survival; PFS, progression-free survival; ICI, immune checkpoint inhibitor; CHT, chemotherapy; PD-L1, programmed death-ligand 1.

**Table 5:** Multivariable logistic regression analysis of factors associated with treatment-related toxicity.

Variable	OR	95% CI	*p*
Vaccination (≥2 vs. 0–1 doses)	2.47	1.06–5.76	0.037
Age (continuous)	1.01	0.96–1.06	0.739
Treatment (CHT + ICI vs. ICI alone)	4.94	0.97–25.24	0.055
PD-L1 expression	0.52	0.23–1.20	0.127
Metastatic burden (>3 vs. ≤3 sites)	0.82	0.38–1.79	0.622

OR, odds ratio; CI, confidence interval; CHT, chemotherapy; ICI, immune checkpoint inhibitor. Outcome: occurrence of any-grade treatment-related adverse event, modelled as a binary ever/never variable without standardization to duration of treatment exposure. All variables were entered simultaneously into a single multivariable logistic regression model. This analysis was exploratory and was not corrected for multiple comparisons.

## Data Availability

The data supporting the findings of this study are not publicly available due to privacy and ethical restrictions, as they contain information that could compromise the privacy of research participants. De-identified data may be made available by the corresponding author upon reasonable request and with permission from the Institutional Ethics Committee.

## Abbreviations

CI	Confidence interval
CTCAE	Common Terminology Criteria for Adverse Events
ECOG	Eastern Cooperative Oncology Group
HR	Hazard ratio
ICI	Immune checkpoint inhibitor
irAE	Immune-related adverse event
mRNA	Messenger ribonucleic acid
NGS	Next-generation sequencing
NSCLC	Non-small cell lung cancer
ORR	Objective response rate
OS	Overall survival
PD-1	Programmed cell death protein 1
PD-L1	Programmed death-ligand 1
PFS	Progression-free survival
RECIST	Response Evaluation Criteria in Solid Tumors
SARS-CoV-2	Severe acute respiratory syndrome coronavirus 2
TPS	Tumor proportion score
